# New Modalities of 3D Pluripotent Stem Cell-Based Assays in Cardiovascular Toxicity

**DOI:** 10.3389/fphar.2021.603016

**Published:** 2021-03-29

**Authors:** Barbara Orsolits, Zsófia Kovács, János Kriston-Vizi, Béla Merkely, Gábor Földes

**Affiliations:** ^1^ Heart and Vascular Center, Semmelweis University Budapest, Budapest, Hungary; ^2^ Bioinformatics Image Core (BIONIC), MRC Laboratory for Molecular Cell Biology, University College London, London, United Kingdom; ^3^ National Heart and Lung Institute, Imperial Centre for Experimental and Translational Medicine, Imperial College London, London, United Kingdom

**Keywords:** human induced pluripotent stem cells, cardiovascular, 3D models, assay, toxicology

## Abstract

The substantial progress of the human induced pluripotent stem cell (hiPSC) technologies over the last decade has provided us with new opportunities for cardiovascular drug discovery, regenerative medicine, and disease modeling. The combination of hiPSC with 3D culture techniques offers numerous advantages for generating and studying physiological and pathophysiological cardiac models. Cells grown in 3D can overcome many limitations of 2D cell cultures and animal models. Furthermore, it enables the investigation in an architecturally appropriate, complex cellular environment *in vitro*. Yet, generation and study of cardiac organoids—which may contain versatile cardiovascular cell types differentiated from hiPSC—remain a challenge. The large-scale and high-throughput applications require accurate and standardised models with highly automated processes in culturing, imaging and data collection. Besides the compound spatial structure of organoids, their biological processes also possess different temporal dynamics which require other methods and technologies to detect them. In this review, we summarise the possibilities and challenges of acquiring relevant information from 3D cardiovascular models. We focus on the opportunities during different time-scale processes in dynamic pharmacological experiments and discuss the putative steps toward one-size-fits-all assays.

## Preserving or Rebuilding Cardiovascular Structures for Modelling

**FIGURE 1:**
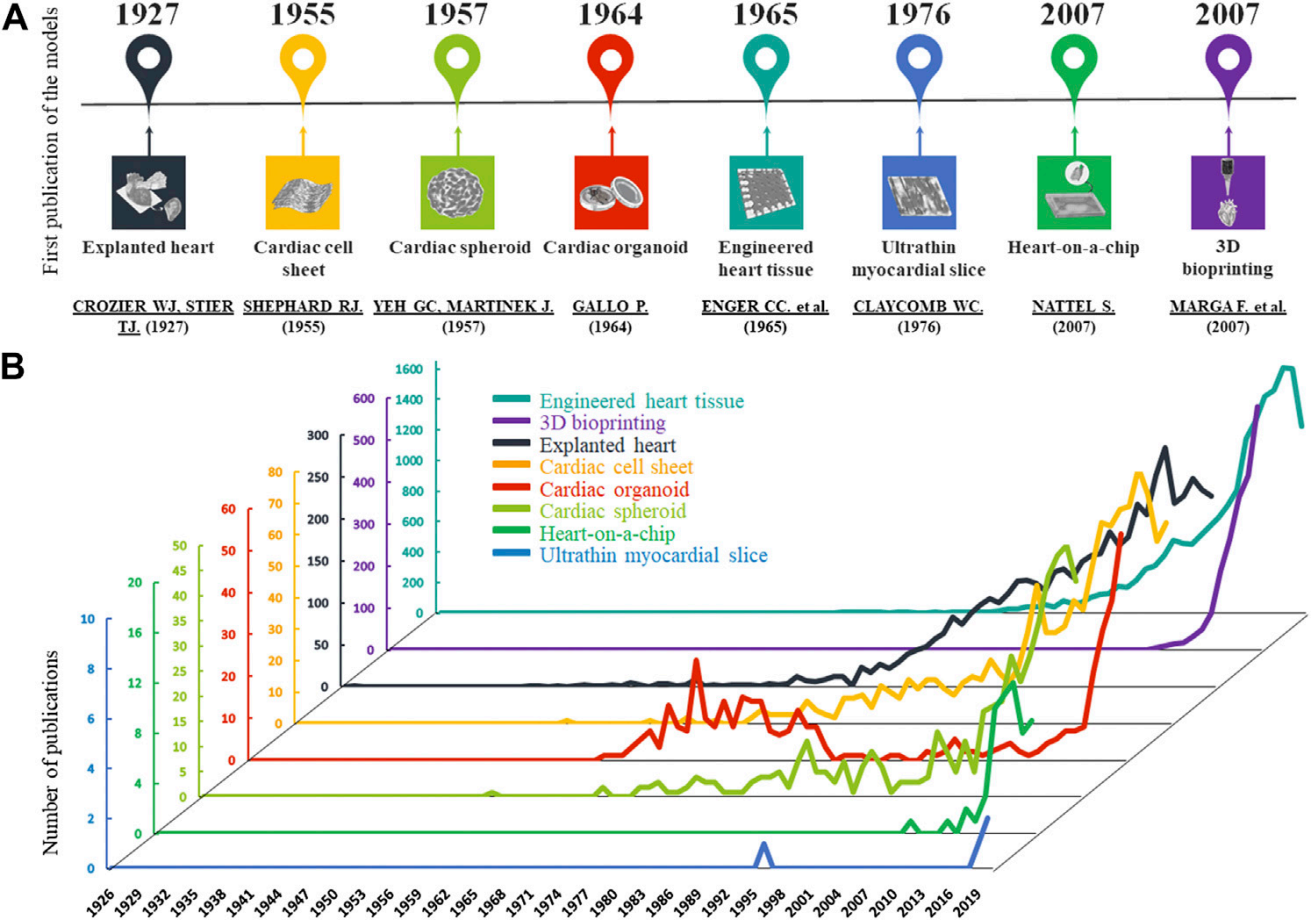
Development of cardiac models. **(A)**: The graphs show the number of published articles of the different models on a timeline based on PubMed data. In the last 20 years, the number of publications has increased in all cases. **(B)**: The timescale presents the first publications of the cardiac models based on PubMed.

The human heart is a complex organ with multiple cell types, including cardiomyocytes, fibroblasts, endothelial cells and perivascular cells, scaffolded by extracellular matrix (ECM) (Pinto et al., 2016). Functional and structural changes occur during cardiovascular disease development, presented with different spatial and temporal dynamics. Thus, there is an unmet need for complex 3D models with realistic architecture which can mimic physiological and pathophysiological conditions. Compared with conventional 2D cell cultures, the construction of organ-like cardiac 3D models may provide a higher fidelity system to investigate cell function and viability. Novel 2D and 3D cell and tissue culture techniques are being developed for disease modeling, drug discovery and toxicity testing. The first 3D cardiovascular models appeared about 100 years ago. Yet, the importance of these technologies has increased only for the last decade. The advancement and the increasing number in publications of these cardiac models are shown in Figure 1.

One of the first approaches is the use of myocardium of the explanted hearts from animals or patients which preserve the original architecture. One can also gather samples during interventions such as coronary artery bypass surgery, valve replacement, closure of ventricular or atrial septal defects and cardiomyotomy (Lal et al., 2015). These serve as excellent sources of ethically-sourced myocardial samples of end-stage failing hearts. Their particular advantage is that the donor’s medical history, the clinical presentation of the disease, and the genetic background are usually available. Examination of explanted heart samples represents a platform for identifying and optimising heart disease treatment strategies and developing better diagnostic tools (Song et al., 2014). However, this approach is not suitable to handle a large number of samples (Tudorache et al., 2013) or for long-term experiments (Harding et al., 1992). These limitations explain why explanted hearts may be inadequate for routine toxicological testing. The advantages and disadvantages of this and other cardiac models below are summarised in Table 1.

**TABLE 1:** Advantages and disadvantages of technologies in cardiovascular modeling.

Technologies	Advantages	Disadvantages	References
Explanted heats	Preserve the original architecture of the organ.	Ethical concerns, time limitation for measurements.	Song et al., (2014)
			Hartung and Corsini (2013)
Ultrathin myocardial slices	Several slices can be prepared from each organ, preserves tissue structure, appropriate model for acute pharmacological testing and in vitro safety screening, ensures oxygen diffusion.	Requires a novel protocol for culturing myocardial slices in their naative state, with preserved structure and function.	Pitoulis et al., (2020)
			Watson et al., (2019)
Engineered heart tissues	Sarcomeric alignment of CMS, improved contractile function, electromechanical cellular coupling.	Requires optimization, lack of vasculature, challenges with cells or materials, issues with tissue maturation, hurdles of imaging.	Eder and Herrling (2016)
			Eng et al., (2013)
			Zimmermann et al., (2004)
Spheroids	Co-culture ability, high reproducibility, inexpensive and less labor intensive than animal models, adaptable for medium-to-high throughput applications.	Size variability and non uniformity of spheroids, limited diffusion, limitation of complexity, small seeding number of cell is challenging.	Mehta et al., (2012)
Cell sheets	Possibility to make various tissue reconstructions such as cardiac patches.	Poor nutrition, hypoxia, necrosis can occur in the middle of multilayered cell sheets.	Chen et al., (2015)
			Yamato and Okano (2004)
Organoids	Biomimetic microenvironment, ability for long term culture, models for translational medicine.	Non-efficient testing platform, must be optimized, imaging challenges, shape of organoids changes constantly.	Richards et al., (2020)
			Nugraha et al., (2018)
			Hynds and Adam (2014)
Organ-on-a-chip	Constant nutrition, oxygen supply and waste removal, in vitro drug screening, mimics dynamic physical vascular microenvironment, tissue-tissue interfaces, vascular perfusion, high throughput screening, long-term co-culture.	Variation and inconsistency between different manufacturing batches, laminar flow causes poor mixing, requires integration of measuring systems.	Ingber (2018)
			Ribas et al., (2016)
3D bioprinting	Fully automated method, ability to bioprint 3D structure of heart.	No functional performance or histological data, immature and improper mirocirculation due to inadeuate perfusion.	Birla and Williams (2020)

### Ultrathin Myocardial Slices

Myocardial slices are ∼100–400 μm slices of living adult heart and can also be used as a model for the native myocardium. These ultrathin slices preserve native cardiac contractility, physiology and its complex multicellular structure. Protocols are being optimised for prolonged survival of these tissue slices in their native form (Watson et al., 2019). The most challenging issue is how to prepare these slices while preserving the native tissue architecture. Manual slicing has been replaced by vibratomes over the decades, making it easier to produce them and be suitable for drug and toxicology testing (Pitoulis et al., 2020). Slices can be obtained from animals and human biopsies. The main advantage is that the preparation is scalable: one can get multiple slices from each heart, allowing one to simultaneously carry out several tests. Various assays have been created to provide high-resolution real-time monitoring of changes in the function and architecture of the slices. Based on laser diffraction, the length of the sarcomeres can be detected as a functional surrogate for contractility and Ca^2+^ signaling. These myocardial slices can be used for *in vitro* drug testing as they still show reproducible pharmacodynamics even beyond 24 h after preparation (Bussek et al., 2012). Cardiotoxic compounds like doxorubicin and allylamine were tested on rat myocardial slices, which reduced protein synthesis and ATP content and increased lipid peroxidation (Parrish et al., 1994).

In addition to human myocardium, with the discovery of human induced pluripotent (hiPSC) technologies, there may be a new source of authentic human cells at hand to find accurate and specific answers in cardiovascular pharmacology. The reprogramming human somatic cells into hiPSC, and their subsequent differentiation to any cell types, allows the production of personalised, well-characterised cardiomyocytes and other cardiovascular cell derivatives, in expandable and long-term cultures. Furthermore, with the use of hiPSC, we can overcome some of the ethical concerns that have plagued human embryonic stem cell and animal models (King and Jacob, 2014; Volarevic et al., 2018). We can also eliminate the species differences and perform conventional higher fidelity human-specific *in vitro* assays. Finally, by combining iPSC and gene editing technologies (i.e. CRISPR/Cas9) one can create cells with a well-defined genetic background (Boretto et al., 2019; Gopal et al., 2020; Sacchetto et al., 2020). Human iPSC cultures can remain genetically and phenotypically stable for long periods, permitting us to monitor time-dependent processes during the maintenance (Liu et al., 2020). Human iPSC allow investigations of heart disease-associated models *in vitro*. Seeding hiPSC-CM (hiPSC-derived cardiomyocytes) onto thin decellularised thin myocardial slices created tissue-like constructs that exhibit a robust response to cardiac drugs for a wide range of concentrations and pacing rates. These recellularised slices recapitulated structural, functional and electrophysiological features of native myocardium and proved sensitive in drug screening assays (Blazeski et al., 2019). Despite the advantages, ultrathin myocardial slices are not yet suitable for chronic experiments, this limits their use in hiPSC-related drug discovery and complex toxicology studies.

### Cell Sheet Engineering

Cell sheet technology is a scaffold-free method where thermosensitive substrates produce single cells and complete layers of cells for tissue regeneration. This cardiac tissue engineering approach may provide better heart models in the long-term (Sekine et al., 2016; Kobayashi et al., 2019; Zurina et al., 2020). The technique generally uses specific culture dishes, coated with temperature-sensitive polymers such as poly(N-isopropylacrylamide). It allows the cells to adhere, spread and proliferate at 37°C. However, at 32°C, the polymer dissolves in water, and the cells spontaneously detach from the culture. This method’s advantage is that adherent cells can be harvested without using any proteolytic enzymes, severely damaging the cultured cells (Yang et al., 2005). This way, the cell adhesion molecules of the cultured cells and the cell-cell junction proteins can be preserved, and the extracellular matrix remains deposited in the cell sheets without degradation. This technology is readily usable, and live cell sheets can be generated. For hiPSC-CM models, the “Cardiac *In Vitro* Proarrhythmia Assessment initiative” may be the first attempt for drug proarrhythmic potential assessment on cell sheets (Gintant et al., 2019).

### Spheroids

The ample literature of 3D spheroid cell culturing spans decades (Figure 1). Spheroids are adherent cell populations which organise in a spherical shape. They can be formed by various scaffold-free methods like spinner flasks, hanging drops, or non-adhesive surfaces. There is a continuous agitation in spinner flasks that prevents the sedimentation of the cells to the bottom of the flask and promotes cell-cell adhesion to form spheroids in suspension. This method allows for long-term cell culturing with sufficient nutrient supply (Mehta et al., 2012). This technique’s initial disadvantages had been the non-uniformly created spheroids, and the undesirable shear stress generated during the continuous agitation (Lin and Chang, 2008). Indeed, these methods mostly produced spheroids which are variable in size, complexity and morphology. Spheroids could only reach a few hundred micrometres in size due to the limited oxygen and nutrient distribution. Thus, for *in vitro* testing, particularly for drug screening, we needed to improve the quality and increase the predictive power of these 3D cultures (Fennema et al., 2013).

3D spheroids were studied in a low-throughput fashion so far. Seemingly trivial components of an image acquisition pipeline, such as identifying the 3D spatial location of a spheroid in an ECM volume, the localisation challenge, becomes prohibitively complex as soon as human expertise is needed to be replaced with robotic automation. The first label-free, standardised, 96-well plate-based systems that enable the pharmacological responses of 3D hiPSC-CM spheroids are now available (Burnham et al., 2020). The high-content analysis of hiPSC-CM serves another example where the individual heart muscle cell locations were identified using image analysis followed by 3D re-imaging with a high magnification lens (Földes et al., 2014). Most commercially available 3D high-content imaging systems are primarily designed to acquire adherent monolayer cell images in specified field-of-view locations in wells. However, a 3D spheroid cultured inside ECM can be located randomly in its volume (Debnath et al., 2003). The spatial localisation challenge of 3D cardiovascular spheroid imaging can be addressed using a non-adhesive, ultra-low attachment surface combined with round bottom well geometry to ensure the spheroid’s central positioning gravitational force. The use of the same multiwell plate for both culturing and imaging of the spheroids simplifies the workflow, eases the spheroid maintenance and reproducibility (Bresciani et al., 2019) as increases the throughput and at the same time decreases the spheroid size and shape variability. The stiff plastic can affect cell physiology, and the long culturing time increases the chance of edge effects. Live imaging of spheroids allows viability assessment (LaBarbera et al., 2012). Treatment can be performed directly, adding medium into the wells, and the central location of spheroids allows more straightforward image acquisition (Bresciani et al., 2019). Depending on the feature of interest, we can choose widefield or confocal imaging. In confocal microscopy, Z-stack or single slice imaging can be performed with ∼100 µm maximal penetration depth. The image data size of a 3D time-lapse can easily reach terabyte-scale (Hoffman et al., 2017). However, 3D culturing is not always coupled with Z-stack imaging. As an example, necessary information for spheroid stress gradient studies (metabolic, hypoxic) can be obtained by single optical slice confocal imaging, that intersects the tested spheroid region. We should always minimise the amount of acquired image data using single optical slice confocal imaging as long as it addresses the given question. That principle applies to high-content analysis. Volumetric, surface, or 3D distance feature extraction needs 3D high-throughput image analysis (Boutin et al., 2018) using software equipped with a 26-neighbour connected component analysis algorithm (Hoffman et al., 2017). Otherwise, various image analysis Z-projection methods (maximum, sum, average intensity), can be applied to a 3D Z-stack to reduce the dimensionality and use a wide range of 2D image analysis algorithms.

### Engineered Heart Tissue

Tissue engineering is a relatively new field which combines cells with efficient regenerative capacity, synthetic or native scaffolds, and growth factors to improve the regeneration of injured tissues. Human iPSC-CM can be combined with a hydrogel-based scaffold to recapitulate human myocardium. Characterisation of these engineered heart tissues (EHT) reveals an anisotropic muscle structure, with embedded hiPSC-CM showing more mature structural and contractile properties than those cells in 2D cultures (Eschenhagen et al., 1997). In contrast to 2D cultures, EHT provides a suitable platform to measure contractile properties such as beating rate, contractile kinetics, and force (Eder and Herrling, 2016). Recent advancements in the EHT method have been significant with cell constructions, scaffold modification, in silico analysis or electrical stimulation (Hirt et al., 2012; Eder et al., 2014). However, the dynamic imaging of EHTs remains a hurdle to overcome. With confocal laser scanning microscopy after a tissue clearing, the organisation and orientation of hiPSC-CM can be imaged with sufficient resolution (Nakane et al., 2020). However, a faster confocal imaging system could further improve larger tissue samples imaging in a time- and cost-efficient way.

### Organoids

Cardiac organoids are built from multiple, cardiovascular cell types which can self-organise into an authentic structure with added or self-generated matrices. They show realistic cardiac microanatomy, cell-to-cell and cell-to-matrix interactions, and tissue-specific architecture. Therefore, the creation of patient-specific organoids from hiPSC may be the most advanced 3D technology to date. The first models with primary cell populations that have been gradually replaced by hiPSC-derivatives as cell sources (Hynds and Adam, 2013). Human iPSC-derived cardiac organoids can model ischemic conditions and drug-induced cardiotoxicity (Richards et al., 2020). Like primary cardiac cells, cardiac organoids show measurable action potential activity, spontaneous beating, and abundant expression of cardiac-specific receptors like ryanodine receptor, L-type calcium channels and proteins such as troponin I, ventricular myosin light chain and atrial myosin light chain (Richards et al., 2017). Self-organising organoids contain a major cell type (usually cardiomyocytes). For higher fidelity use and prevascularisation, they can be co-cultured with heterotypic cell types (hiPSC-derived endothelial cells, smooth muscle cells or pericytes) to model cell-cell interactions and cell states (proliferating, quiescent and apoptotic) of myocardial tissue (reviewed elsewhere, see Nugraha et al., 2018; Gomez et al., 2018; Talman and Kivelä, 2018; Nugraha et al., 2019; Madeddu and Foldes, 2019). In multicellular organoids, cell-type-specific post-translational modifications regulate critical biological processes and are frequently dysregulated in disease. Similar to other 3D models, low-dimensional fluorescent imaging cannot capture the complexity of these signaling network nodes. A promising method to comprehensively analyze cell-type-specific changes utilises thiol-reactive organoid barcoding *in situ*. Integrating single-cell post-translational modifications analysis with multiplexed organoid-barcoding enables high-throughput comparison of signaling networks between hiPSC-based heterocellular organoid cultures (Qin et al., 2020). Therefore, hiPSC-derived cardiovascular organoids are an excellent choice for patient-specific toxicity studies; yet, the lack of standardised organoids production results in variable responsiveness to particular treatments (Takebe et al., 2013).

### Heart-on-A-Chip

The organ-on-a-chip is a miniature organotypic cell culture on a chip equipped with a complete microfluidic system. Due to the regulation of microfluidic parameters, we can manipulate the cellular microenvironment and control mini-organ behavior. This complex platform provides constant nutrition and oxygen supply and simultaneous waste removal by its multi-microchannels (which mimics the vascular system). Many single and multiorgan organ-on-chips have been presented (Lee and Sung, 2017; Wu et al., 2020). With fast engineering and biomaterial technology progression, we may even achieve complete human-on-chip technology soon (Marx et al., 2020). Heart-on-chip is an excellent implementation in toxicology by incorporating beating hiPSC-CM (Abulaiti et al., 2020). By high-speed impedance detection on heart-on-chip, we can detect the drug responsiveness on the cardiac tissue (Zhang et al., 2016). Upon drug-related stimuli such as doxorubicin or isoproterenol, the contractile function of the 3D cardiac construct can be detected by the piezoelectric sensing system. At the same time, image processing can provide *in situ* multi-site detections. The amplitude of the voltage output decreases in both treatments, while the contraction frequency is increased by isoproterenol and decreased by doxorubicin. These two measurements can provide us with mutual information of the fabricated heart construct’s contractile behavior on a chip (Sakamiya et al., 2020). For localised real-time monitoring of cellular activities and physicochemical changes in an organ-on-chip, electrical sensors were first integrated with macroporous 3D scaffolds a decade ago (Tian et al., 2012). Functionalised synthetic 3D biomaterials allow for studies of cell/tissue development in the presence of biochemical stimuli and monitoring the pharmacological responses of cells within synthetic tissues may provide a more robust link to *in vivo* disease treatment compared with 2D cell cultures. Seamless integration of 3D (silicon nanowire field effect transistor-based) nanoelectronic scaffolds into tissue materials can serve as lab-on-a-chip pharmacological platforms and records both extracellular and intracellular signals of contracting cardiomyocytes and other cells with subcellular and sub-millisecond time resolution. As an initial example of a drug screening assay, recordings from the nanowire of a 3D cardiomyocyte mesh construct showed increased beating rate in response to adrenergic norepinephrine. Functional tubular structures of vascular nanoelectronics with human aortic smooth muscle cells also showed contractile activity (Marsano et al., 2016).

### 3D Bioprinting

Printing biocompatible materials and supporting components with the desired cells offers us the ability to create precisely designed structures of different cardiac tissue constructs. 3D bioprinting shows extraordinary versatility to build living cardiac tissues in a point-by-point and/or layer-by-layer manner. To date, several constructs have been made available for vascular (Norotte et al., 2009; Duan et al., 2013; Cui et al., 2019), or cardiac use (Cui et al., 2018; Noor et al., 2019). Human iPSC-derived cardiac patches with contractile function have successfully been printed (Lee et al., 2019). High throughput 3D printing of prevascularised hiPSC-CM structures could be personalised for drug discovery and toxicology (Arai et al., 2020). One may envisage that the generation of a subpopulation of chamber-specific cardiomyocytes or arterial/venous endothelial cells would further reduce variability and cellular heterogeneity of the printed constructs.

## Catching Fast-Changing Processes

**FIGURE 2:**
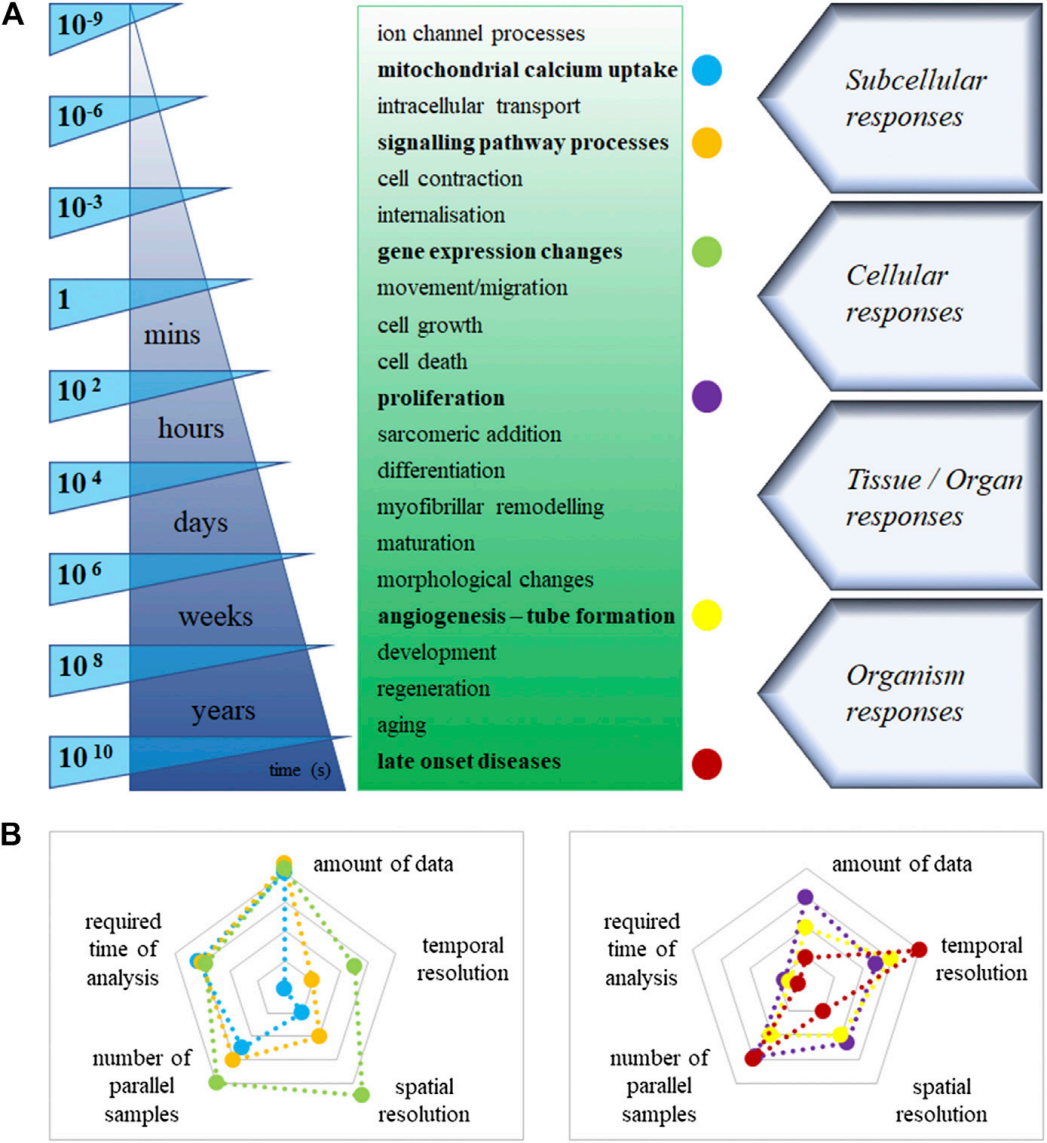
**(A)**: Estimated timeline of the main cellular processes in cardiovascular cells. **(B)**: Labeled cellular processes [colored dots in Panel. **(A)**], displayed on two-dimensional spider charts. The logarithmic scales demonstrate the values of different technical necessities, increasingly from the centre to the edges in the graph.

Changes of cellular and extracellular processes in primary and hiPSC-derived cardiovascular cell constructs are highly dynamic and show variable kinetic profiles (Figure 2). This improved technology with physiologically relevant cellular function data can generate adequate information on pharmacokinetics and pharmacodynamics during drug screening. It may mimic dosing regimens used *in vivo* (similar to insulin regimen in diabetes, natriuretic peptide release in heart failure, as well as endocrinology-related disorders) (Kriston-Vizi et al., 2017). Until recently, the high-throughput imaging methods and reconstruction of images were too slow in many dynamic live-hPSC-derived cell applications, such as the measurements of mitochondrial calcium uptake, sarcomeric addition and myofibrillar remodeling of hiPSC-CM and endothelial cells, lipid droplet formation in metabolic disorders, and internalisation for drug delivery. The recent introduction of commercially available HCS systems (e.g. Hamamatsu Photonics, Molecular Devices) can address fast-response assays, such as calcium flux or cardiomyocyte beating, using imaging frame rates as fast as 100 frames per second (e.g. PerkinElmer). The high temporal resolution allows us to study rapid intracellular processes that happen on a millisecond timescale. Providing multiple endpoints is an excellent advantage of novel high-content imaging-based assays. However, parallel assessment of complex changes in hiPSC-derived cardiovascular cells, such as 3D rearrangements of sarcomere structure and dynamic translocation of natriuretic peptides, transcription factors and other second messengers, requires 3D imaging, high-performance computing and storage. These improved assays can readily predict molecular targets and off-target effects of test compounds, such as cytotoxicity or cardiovascular cell morphology changes. We can better investigate these predominantly short-term manifestations of cardiac pathologies in hiPSC-CM, including assays on depressed cell contraction, changes in electrophysiology, metabolism and intracellular Cafn^++^ levels.

## Long-Term Processes in the Cardiovascular System

Underlying cellular pathways in cardiovascular cell growth, proliferation, survival, and death are controlled by the steady-state level of regulatory proteins and enzymes (Figure 2). Multiple endpoints from high-content assays would provide users with a great advantage to better understand these complex processes. Improved assays can readily predict molecular targets and off-target effects of test compounds, such as cell morphology and viability changes. Indeed, longer-term assays focus on abnormal morphology, hypertrophy and increased susceptibility to cell death. Cell-based assays performed using an automated fluorescence imaging platform, and high-content analysis is valuable in characterising hypertrophic states induced in hiPSC-CM upon exposure to cardiotoxic compounds. We can assess the detailed 3D hypertrophic profiles of cardiomyocytes based on information captured at cellular and subcellular levels. Therefore, the assays can easily predict molecular targets and off-target effects of test compounds, such as cytotoxicity in cardiovascular cell morphology changes. Human iPSC-derived cardiomyocytes and endothelial cells retain their proliferative activity in culture. Compared to conventional static immunohistochemistry, time-lapse imaging, e.g. Premo Fucci system can reveal comprehensive data and could visualise real-time cell cycle transitions (Yano and Hoffman, 2018). Another notable example is the endothelial-mesenchymal transition, a particular version of the vascular system’s epithelial-mesenchymal transition. It is extensively investigated in physiologic cardiac development and pathologic fibrosis. It is accompanied by cell morphology and identity changes in various diseases and one-way drift from one cell type to another with intermediate phases. Similarly, image processing is not currently possible in live assays monitoring tube formation as markers of disease (i.e. angiogenesis for anti-cancer indications). Understanding chronic treatment regimens (rather just one-off curative, like advanced therapies) leveraging *in vitro* platforms may require long-term cultured cells and follow-up, and long-term recording (Habeler et al., 2009).

## Towards Mapping Organoids With High Spatiotemporal Resolutions

Understanding the development, function and pathology of heterogeneous cell-cell interaction within organoids requires system-level mapping of the cell-based activities with high resolutions in space and time across the 3D volumes over a particular time window. Indeed, current workflows rely on fixed cells and can only assess only one time point, not continuous periods. Tissue-wide electrophysiology with single-cell/subcellular spatial and millisecond temporal resolution is critical for high-fidelity and long-term cardiovascular studies. However, it is an issue to invasively implant and localise sensors without destroying the well-connected cellular networks within the complex and matured organoids. Human cardiac cyborg organoids via organogenetic 2D–3D reconfiguration may overcome this hurdle and generate electrophysiological patterns during organogenesis. The platform is also scalable for integrating a larger number of sensors and stimulators fabricated into a stretchable nanoelectric mesh structure. The connection between electronics and cells enables long-term recording of electric dynamics in human cardiac organoids. It may be applicable for hiPSC-based modeling of cardiac diseases and therapeutics (Lancaster and Knoblich, 2014; Li et al., 2019). An alternative approach leverages radio frequency identification (RFID) technology, which is used to trace and track individual objects in multiple contexts by wirelessly providing digital signals; hiPSC-derived organoids integrating RFID microchips inside can be used for phenotypic screens and deliver readouts in real-time via a coiled antenna. Following an aggregation via a self-assembling cavitation process, chips integrate well into the organoids without impairing structure or functions. One proof-of-principle example to prove this was to test fat accumulation using fluorescence imaging based on a phenotyping assay coupled with the RFID-integrated organoids in an inherited hepatic lipid-storage disease model (Kimura et al., 2018). It is again a scalable technology which may become accessible to study genotype-phenotype relations in human cardiovascular pathologies.

## Future Prospects

Drug development and toxicological testing are time-consuming and costly processes and require many parallel samples. To collect more and more information from many parallel samples and complex architectures of our modeling system massively increases the data acquired. Furthermore, the large amount of data increases the complexity of the analysis and the interpretation’s difficulty. Additionally, the high similarity to native organs causes an increase of the initially high financial inputs. Fluorescence microscopy with automated, physiologically relevant cell-based 3D assays and image analysis; immediate identification and recording of cell-based activity; ability to identify novel complex cellular processes by multiple pathways; high-performance computing, storage, analysis and data management; establishment of standardised, high-fidelity *in vitro* models and the large-scale automation in several processes are the key to the new generation of high-throughput toxicological testing with hiPSC derivatives.

There is a hope that these 3D humanised cell platforms will improve predictive capabilities since cardiotoxicity is one of the most common causes for attrition of both cardiac and non-cardiac drugs. Recent advances in generating high-fidelity, *in vivo*-like cellular settings with hiPSC derivatives can provide us with consistent performance while permitting continuous and quantitative imaging. There is a need for better and more affordable medicines, with the burden of disease rising faster than GDP due to an ageing population and increasing chronic disease prevalence. Of importance, cardiovascular disease is the leading cause of mortality and morbidity. Today, less than 30% of all the work in personalised medicine targets the various non-oncological targets. The survey/study like that of the BioIndustry Association confirms the need for competitive improvements in screening technology and processes for humanised stem cell models of drug discovery to enable better predictability in clinical trials. Adaptation of these next-generation technologies could support disease prevention and proactive management of health and chronic conditions. It provides earlier and better detection and diagnosis of disease, leading to better patient outcomes, and finally leads to tailored treatments that either change the underlying disease or offer potential cures. According to the latest report published by FMI, the global organoids market is expected to reach ∼ US$ 134 Mn by the forecast year 2029. The report further projects that the organoids market will grow at a CAGR of ∼15% during 2019–2029 (Valley Cottage, 2019).
