# Supplementary figures and images for: New Modalities of 3D Pluripotent Stem Cell-Based Assays in Cardiovascular Toxicity
Source: Front Pharmacol. 2021 Mar 29;12:603016. doi: 10.3389/fphar.2021.603016 (PMC8039822; doi:10.3389/fphar.2021.603016)

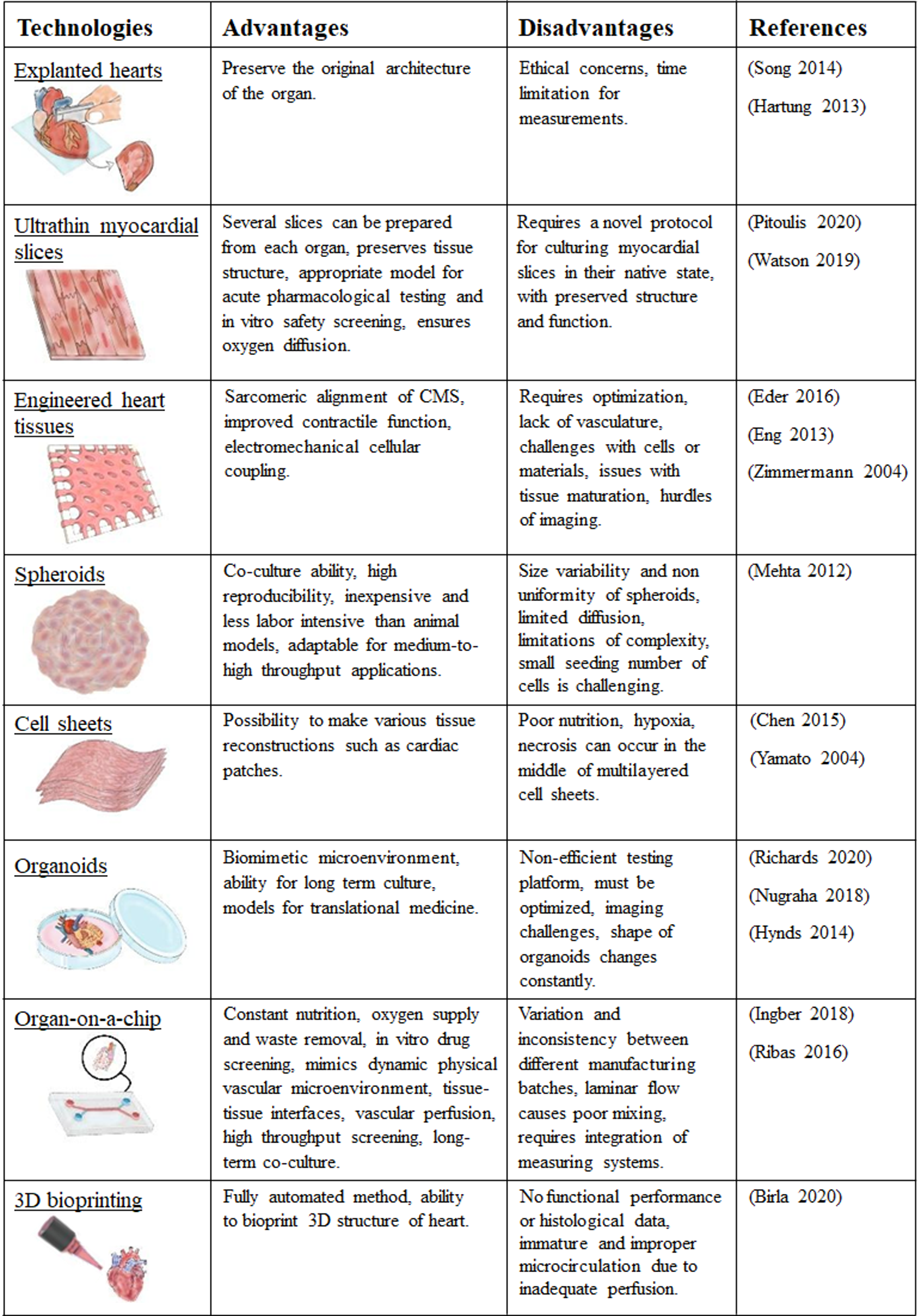

Supplement: Supplementary file 1 [file image1.tif]
